# Cluster-randomized implementation trial of two facilitation strategies to implement a novel information and communications technology at the Veterans Health Administration

**DOI:** 10.1186/s13012-023-01329-5

**Published:** 2024-01-02

**Authors:** Chelsea Leonard, Evan Carey, Ariel Holstein, P. Michael Ho, Jeffrey T. Heckman

**Affiliations:** 1 Denver-Seattle Center of Innovation for Veteran-Centered & Value-Driven Care (COIN), VA Eastern Colorado Healthcare System, Aurora, CO 80045 USA; 2 Division of Health Care Policy and Research, University of Colorado Anschutz Medical Campus, Aurora, CO USA; 3 VA Collaborative Evaluation Center (VACE), Aurora, CO USA; 4 Department of Biostatistics and Informatics, Colorado School of Public Health, University of Colorado Anschutz Medical Campus, Aurora, CO USA; 5 Cardiology Section, Rocky Mountain Regional VA Medical Center, Aurora, CO USA; 6 Physical Medicine & Rehabilitation Service, James A. Haley Veterans’ Hospital and Clinics, Tampa, FL USA; 7 Department of Neurology, University of South Florida Morsani College of Medicine, Tampa, FL USA

**Keywords:** Information and communication technology, Implementation, Facilitation

## Abstract

**Background:**

Information and communication technologies (ICTs) improve quality and efficiency of healthcare, but effective practices for implementing new ICTs are unknown. From 2019 to 2021, the Veterans Health Administration (VHA) implemented FLOW3, an ICT that facilitates prosthetic limb care. The goal of this study was to compare the impact of two facilitation strategies on FLOW3 adoption, implementation, and sustainment.

**Methods:**

FLOW3 is a computerized workflow management system comprised of three applications that facilitate the three steps for prosthesis authorization. During VHA’s implementation of FLOW3, we randomized 60 VHA sites to basic or enhanced facilitation groups. Basic facilitation included a manualized training toolkit and office hours. Enhanced facilitation included basic facilitation plus monthly learning collaboratives and site-specific performance reports. Outcomes included time to adoption of FLOW3 and complete FLOW3 utilization rates during implementation and sustainment periods. We compared outcomes between sites assigned to basic versus enhanced facilitation groups. Results were calculated using both intent-to-treat (ITT) and dose–response analyses. The dose–response analysis used a per-protocol approach and required sites in the enhanced facilitation group to join two of six learning collaboratives; sites that attended fewer were reassigned to the basic group.

**Results:**

Randomization assigned 30 sites to enhanced facilitation and 30 to basic. Eighteen of 30 randomized sites were included in the enhanced facilitation group for dose–response analysis. During the implementation period, enhanced facilitation sites were significantly more likely to completely utilize FLOW3 than basic facilitation sites (*HR*: 0.17; 95% *CI*: 1.18, 4.53, *p* = 0.02) based on ITT analysis. In the dose–response analysis, the enhanced group was 2.32 (95% *CI*: 1.18, 4.53) times more likely to adopt FLOW3 than basic group (*p* = 0.014).

**Conclusions:**

Enhanced facilitation including a learning collaborative and customized feedback demonstrated greater likelihood for sites to complete a prosthetics consult using FLOW3 throughout our study. We identified statistically significant differences in likelihood of adoption using the dose–response analysis and complete utilization rate using ITT analysis during the implementation period. All sites that implemented FLOW3 demonstrated improvement in completion rate during the sustainment period, but the difference between facilitation groups was not statistically significant. Further study to understand sustainability is warranted.

Contributions to the literature This study reports on one strategy to enhance facilitation during implementation of a novel information and communications technology by incorporating learning collaboratives and providing individualized feedback to improve adoption and increase consistent and comprehensive utilization of the new workflow.

- Personalized feedback and learning collaboratives improve adoption and implementation, while further work to understand continued sustainment of ICT is warranted.
- Comparison of facilitation strategies using intent-to-treat and dose–response analyses is critical to identify the value of participation in the facilitation strategy and its effects.

## Background

Information and communication technologies (ICT) have been shown to improve quality and efficiency of care [1–3], but studies suggest that providers and healthcare systems often struggle to adopt and implement new ICT [4–6]. Barriers and facilitators to implementation of ICT have been previously described [6–11], but to our knowledge, there is no existing literature on evidence-based strategies that facilitate adoption, implementation, and sustainment of novel ICT. There is an urgent need to better understand effective strategies to implement and sustain novel ICT to ensure that patients benefit from these advances.

The Veterans Health Administration (VHA) provides prosthetic limb care for as many as 14,000 veterans with limb loss annually. Veterans receive prosthesis management as part of a team-based interdisciplinary clinic evaluation by rehabilitation providers. Both internal and external reviews identified variability in the VHA prosthetic limb care process as well as overpayments and lack of a comprehensive system to monitor this process [12, 13]. To address these issues, frontline staff developed a novel ICT called FLOW to optimize the prosthetic limb care process and improve value-based prosthetics procurement at VHA. Results during the initial pilot phase revealed a greater than 2-week improvement in efficiency for authorization of an artificial limb. VHA policy makers identified this significant process improvement and published a memorandum supporting implementation throughout the VA on September 27, 2018. Through iterative design and development, the third version of FLOW, FLOW3, became a computerized workflow management system that streamlines and standardizes the prosthetic limb care process. FLOW3 consists of three custom designed software applications that guide clinicians and staff through the five steps of the limb provision process. These steps include the following: (1) limb prescription, (2) coding the prescription, (3) authorization, (4) delivery to the patient, and (5) evaluation of fit and function.

To address the gap in evidence regarding effective facilitation strategies for adoption, implementation, and sustainment of ICT innovations, we designed a randomized implementation trial (e.g., [13–20]) of FLOW3 at 60 VHA sites. We compared the impact of “basic” versus “enhanced” approaches to facilitation and audit and feedback [21] on the adoption, implementation, and sustainment of an ICT innovation in a large and diverse healthcare system. Our aims were as follows: (1) describe participation in enhanced versus basic facilitation groups; (2) describe differences in time to adoption, utilization rate of all FLOW3 applications during the implementation period, and sustained use of all FLOW3 applications beyond the implementation period using an intent to treat (ITT) analysis; and (3) describe differences in time to adoption, utilization rate of all FLOW3 applications during the implementation period, and sustained use of all FLOW3 applications beyond the implementation period using a dose–response analysis.

## Methods

### Prosthetic limb care in the VHA

VHA provides inpatient and outpatient clinical care for veterans and active-duty service members with acquired absence of a limb using a multidisciplinary approach at over 150 sites across the USA. This team-based clinical pathway provides comprehensive and lifelong prosthetic limb care with the goal of optimizing functional independence and quality of life. The management of prosthetic limb care requires clinical decision-making and is initiated by a prescription to document diagnosis, medical justification, and specific characteristics of the prosthetic limb. Following prescription entry, the limb order must be specified and authorized for purchase to ensure that the order meets VHA standards for purchasing and contracting. The limb may be fabricated within prosthetic facilities at the Department of Veteran’s Affairs (VA) sites or non-VA prosthetic facilities in the community. The responsibility for authorization of a prosthetic limb is shared among clinical and nonclinical staff members including prosthetists, purchasing agents, and contracting specialists. Once a prosthetic limb is authorized, fabrication of the materials can begin. After the limb is completed and fit and function are confirmed, the limb is delivered to the patient. Finally, clinical follow-up by the prescribing team verifies the quality of the limb and allows for continued coordination of care for rehabilitation services.

### FLOW3 background

**Fig. 1:**
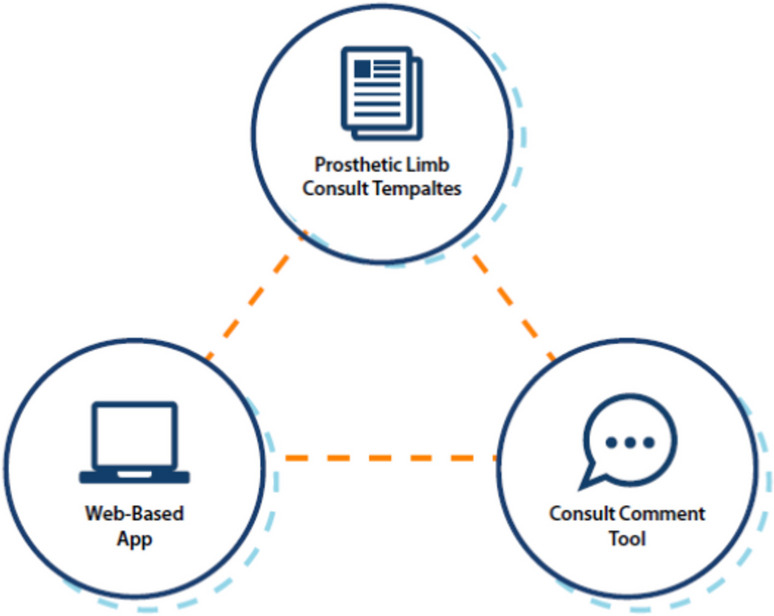
Summary of FLOW3 applications, which include the prosthetic limb consult templates, web-based application, and consult comment tool

FLOW3 was created through an iterative approach by a team of VHA frontline staff members with support of their leadership with the goal of standardizing and streamlining the prosthetic limb care process using the available information technology. FLOW3 is an ICT comprised of three interrelated applications to support the prosthetic limb care process: (1) a prosthetic limb consult template that allows for accurate description of limb specifications, (2) a consult comment tool that allows for multidisciplinary communication and automates delivery of information to the third component, and a (3) web-based application which compiles all information and communications for review and notification of action items related to the care process. Figure 1 summarizes the three FLOW3 applications. FLOW3 connects the staff and clinicians involved in all steps of the prosthetic limb procurement process. Each team member uses at least one of the three applications to move the process forward.

**Fig. 2:**
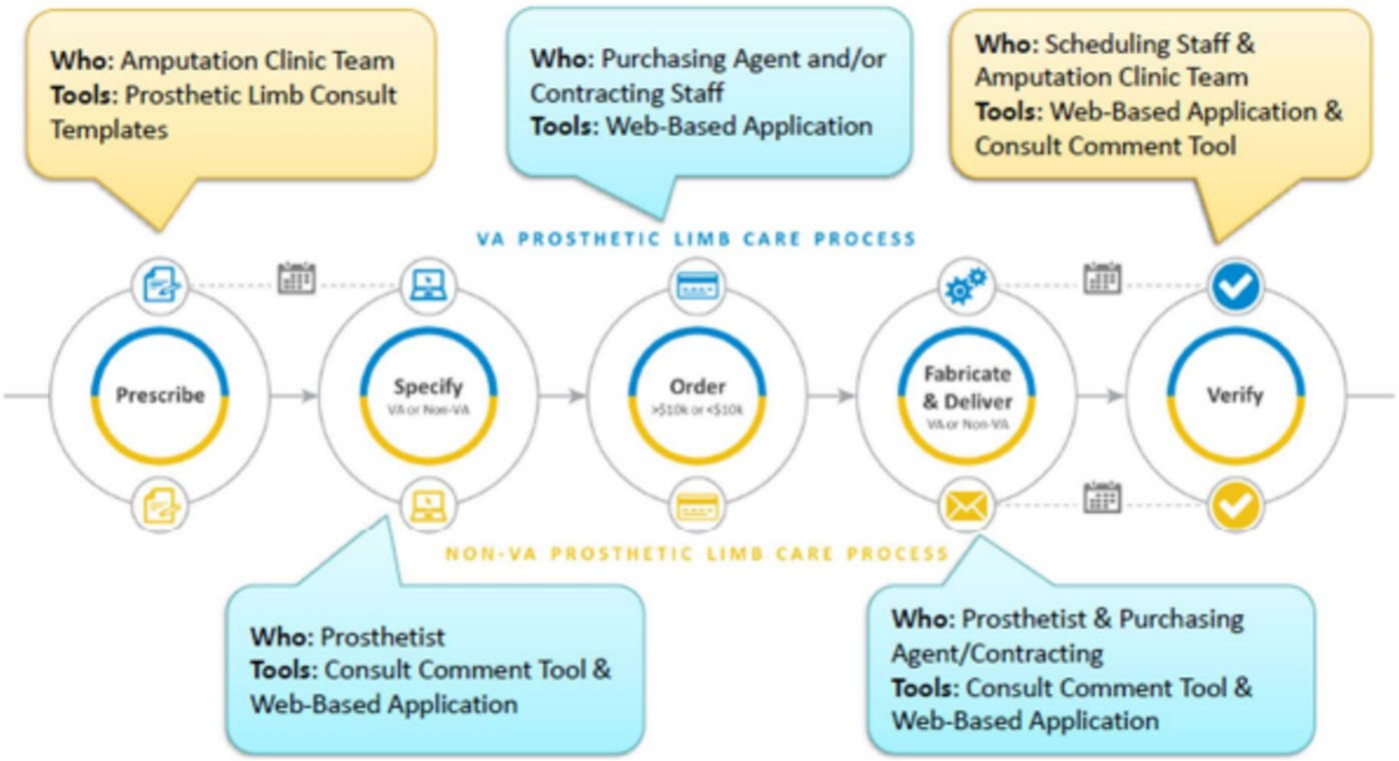
This figure summarizes the limb care process and details the role responsible for each step and the FLOW3 application used

Figure 2 summarizes the limb care process and illustrates how FLOW3 facilitates each step for the staff and clinicians involved. The first FLOW3 application, the prosthetic limb consult template, facilitates the first step of the prosthetic limb care process. In this step (prescribe), a prescription is entered by a licensed independent practitioner into the computerized patient record system (CPRS), the VHA’s electronic health record, using one of four prosthetic prescription templates. The consult comment tool is linked to CPRS and facilitates the second step (specify). In this step, the prescription is described using the Healthcare Common Procedure Coding System (HCPCS) by a certified prosthetist. The web-based application facilitates the third and fourth steps of the process. During the third step (order), the HCPCS list from the consult comment tool is automatically organized into a PDF document with information for authorization of a purchase order and available on a web-based application. The information from this PDF document is accessed by purchasing staff from the web-based application and manually transcribed to purchasing software to create a purchase order authorization. Following purchase order authorization, the fourth step (fabricate & deliver) involves the direct patient care by a prosthetist including initiation of ordering of materials and fabrication activities to complete the prescribed prosthetic limb. This step culminates in fitting and delivery of the limb to the patient. The consult comment tool is used to complete the final step. The fifth step (verify) in the process is completed during follow-up clinical care by an interdisciplinary team evaluation to verify the quality of the limb or determine if any aspect needs to be reworked. Each of these five steps are monitored with date and time stamps allowing for oversight and reporting.

### Trial design

This study was conducted as part of a larger, multi-year evaluation of FLOW3 implementation, system-level impacts, and patient-level impacts guided by the RE-AIM framework [22]. FLOW3 was supported by policy from the Department of Veteran’s Affairs (VA) Rehabilitation and Prosthetics Services towards system-wide implementation across VHA in several phases. In Phase 1, FLOW3 was recognized as a practice that addresses important areas for improvement in veteran care, and it was implemented at eight VA sites in 2017. In Phase 2, FLOW3 continued rollout to an additional 15 VA sites. FLOW3 was selected for national rollout in 2019 and partnered with an evaluation team to facilitate implementation and expansion as it was implemented across VHA. Implementation and program outcomes were evaluated across the first two phases and shared with clinical stakeholders and policy makers. At the time of this study, FLOW3 was already implemented at 23 VA sites. Implementation efforts were led by the “FLOW3 implementation team” consisting of the physician, prosthetist, data programmer, and project manager who led development of FLOW3 and evaluated by an evaluation team consisting of an anthropologist, two health services researchers, data scientist, statistician, programmer, and professional research assistant.

### Participants and setting

Prior to implementation at each site, VA Integrated Service Network (VISN) and VA site leadership were contacted at each of the 60 VA sites selected for Phase 3 of implementation to identify physical medicine and rehabilitation (PM & R) and prosthetic and sensory aid services (PSAS) team members involved in prosthetic limb care. Additionally, leadership was asked to identify VA sites prosthetic limb care “champions” based on the following characteristics: comfort with technology, strong communication skills, and ability to lead training for other staff at their sites. FLOW3 Champions at each VA site assisted in coordination of installation steps for FLOW3 custom software, as well as introducing FLOW3 to their team members. Test environments in the electronic medical record allowed for FLOW3 implementation team members to confirm proper installation of prescription templates and custom software.

### Implementation intervention

The FLOW3 implementation team provided external facilitation to both basic and enhanced facilitation groups. We defined facilitation as ongoing “interactive problem solving and support” [23] provided by the implementation team within the context of FLOW3 implementation. The basic facilitation group had access to monthly office hours with the FLOW3 implementation team and access to an email address where they could send any inquiries about using the system. During office hours, the members of the implementation team were available via Microsoft Teams, and frontline FLOW3 users could join at any point during the 2-h window to troubleshoot issues they were experiencing. Issues could be technical or related to resources and support needed from departments or leadership to support implementation. They did not have to attend the entire duration of the office hours, nor did they have to attend every month.

The enhanced facilitation group was invited to six structured, 1-h monthly calls with the FLOW3 implementation team. For each site, the site champion and at least one other staff member were invited to attend. Each call included an icebreaker, prepared content related to FLOW3, discussion regarding use of FLOW3 at participants’ sites, and time for questions. In addition to this call, site champions at enhanced facilitation sites received performance reports with a monthly action plan via email detailing FLOW3 utilization along with personalized recommendations of how to increase utilization locally. Sites in the enhanced facilitation group also had access to the email address shared by the FLOW3 implementation team to have their questions answered outside of the scheduled monthly meeting.

Differences in time to adoption, implementation, and sustainment of FLOW3 utilization between the basic and enhanced groups were evaluated using both intent-to-treat (ITT) and dose–response analyses.

### Site recruitment

**Fig. 3:**
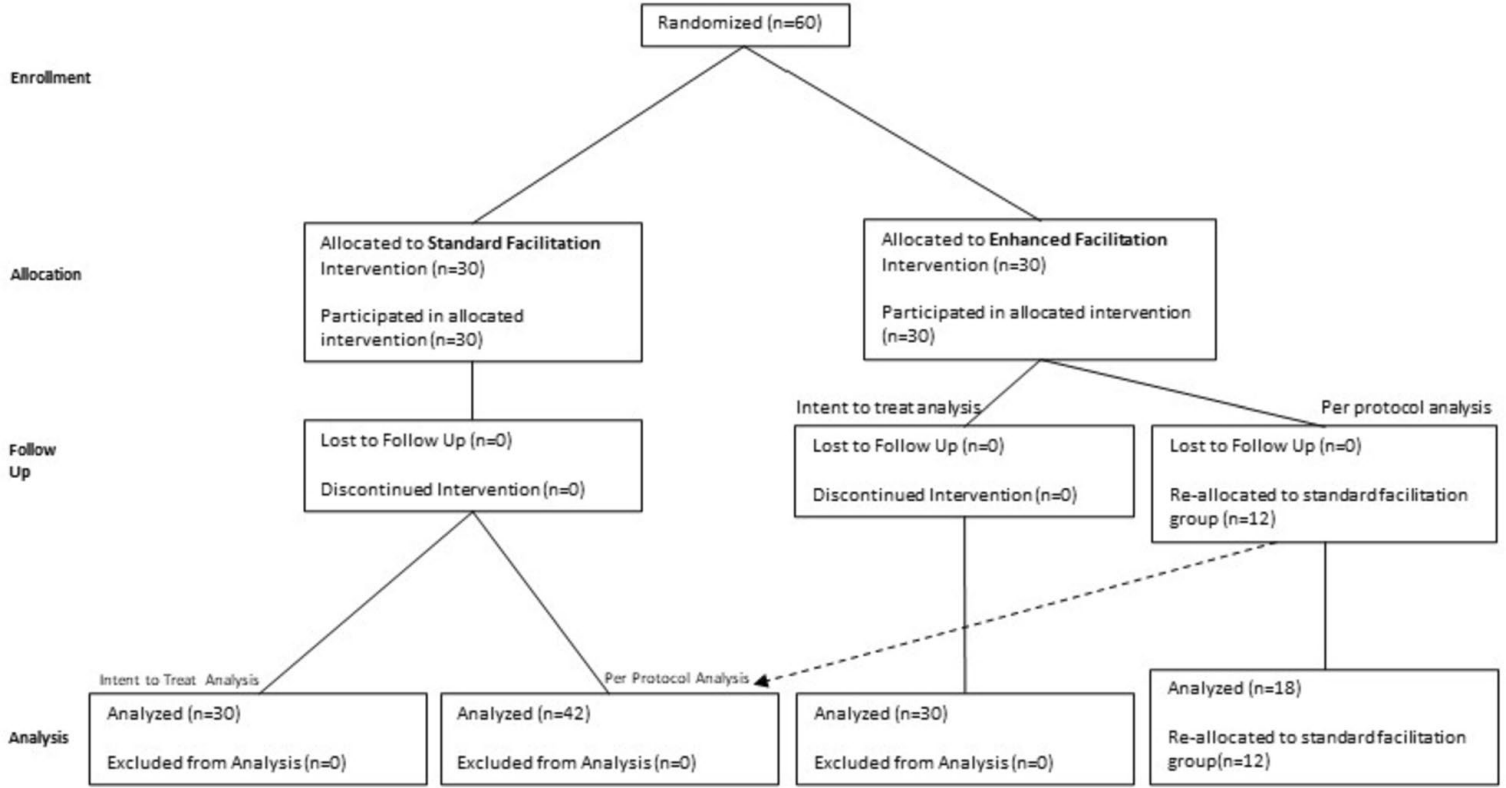
Summarizing study design and recruitment

This study focused on Phase 3 of system-wide implementation of FLOW3 at 60 VA sites between October 29, 2020, and July 30, 2021. The 23 sites in Phases 1 and 2 of implementation were not eligible for this study because FLOW3 was already implemented. The FLOW3 implementation team selected 60 sites for Phase 3 based on the sites’ willingness to implement FLOW3 beginning in October 2020. All 60 Phase 3 sites were included for randomization. This study used a two-arm clustered randomized trial design (Fig. 3).

### Outcome definitions

#### Time to adoption (site level)

We defined adoption as the first prosthetics consult entry at a site that utilized all three FLOW3 applications (Fig. 1) to obtain an authorization. We documented the prescription date of that first consult as the site adoption date. The time to adoption was then defined as the number of days between the launch date (October 29, 2020) for Phase 3 implementation and the site adoption date.

#### Complete utilization rate during implementation period

We looked at this outcome using both consult level and site-level analyses.

##### Consult level analysis

We defined complete FLOW3 utilization as recording of all three FLOW3 components per consult entered at a site: (1) the prescription template, (2) the consult comment tool, and (3) the web-based application. We assessed the percentage of prosthetics consults that demonstrated complete utilization at each site during the 6-month implementation period (October 29, 2020–April 29, 2021). Because this is a consult level analysis, sites with more consults are weighted higher than sites with fewer consults.

##### Site-level analysis

We compared the consult completion rate at each site without weighting by the number of consults placed.

#### Complete utilization rate during sustainment period

We looked at this outcome using both consult level and site-level analyses.

##### Consult level analysis

We evaluated the percentage of consults that demonstrated complete utilization at each site during the 3 months following the implementation period (April 30, 2021–July 30, 2021). Because this is a consult level analysis, sites with more consults are weighted higher than sites with fewer consults.

##### Site-level analysis

We compared the consult completion rate at each site without weighting by the number of consults placed.

### Description of facilities and utilization

To investigate whether our facilitation interventions would affect FLOW3 adoption, implementation, and sustainment at a site level, we first evaluated the number of consults entered since the launch date for Phase 3 implementation (i.e., October 29, 2020) and included these data in a live view database that stored FLOW3 data since October 2017 named FLOW3Raw2.

### Primary analysis—intent-to-treat analysis

We performed three evaluations to compare basic to enhanced facilitation strategies using an intent-to-treat (ITT) analysis [24–26]. First, we evaluated for differences in time to adoption. Then, we evaluated for differences in complete utilization rates of FLOW3 during the implementation period. Finally, we evaluated for differences in complete utilization rates of FLOW3 during the sustainment period. The ITT analysis examined the outcomes for each site based on facilitation group assignment, regardless of whether sites participated in the basic or enhanced facilitation offerings by the FLOW3 implementation team.

### Secondary analysis—dose–response analysis

We performed three evaluations to compare basic to enhanced facilitation strategies using a dose–response analysis. This analysis used a per-protocol (PP) approach to differentiate between sites that participated in the enhanced facilitation protocol versus those that did not participate [24–26]. First, we evaluated for differences in time to adoption. Then, we evaluated for differences in complete utilization rates of FLOW3 during the implementation period. Finally, we evaluated for differences in complete utilization rates of FLOW3 during the sustainment period. Our study team developed our protocol by consensus. The protocol required that sites in the enhanced facilitation group participate in at least two of the monthly learning collaboratives and confirmed receipt of recommendations provided by the FLOW3 implementation team via email or verbally. At the beginning of each monthly call sites were asked to confirm that they received recommendations and given the opportunity to ask questions or brainstorm. Sites that did not meet the minimum threshold for our enhanced facilitation protocol were evaluated in the basic facilitation group. The secondary analysis used the same analytic methods as the primary analysis.

### Randomization

We randomized implementing sites to compare two distinct facilitation strategies: basic and enhanced. We identified whether sites are identified as Polytrauma Amputation Network Sites and receive special funds for staffing a physiatrist, rehabilitation coordinator, and administrative support from the VA Amputation System of Care (ASoC) [27]. Sites were randomized in R using stratified randomization by healthcare system network and ASoC support to ensure equal allocation of exposed and unexposed groups in each healthcare system network. Neither sites nor implementers were blinded to which sites were included in each of the groups.

This project was designated quality improvement by the Colorado Multiple Institutional Review Board (COMIRB) and was therefore exempt from Institutional Research Board review [28]. It was supported by the Quality Enhancement Research Initiative (QUERI), VHA Diffusion of Excellence (DOE), and VHA Rehabilitation and Prosthetic Services (RPS) as part of a partnered evaluation initiative.

### Analytic methods

#### Kaplan–Meier curves and Cox proportional-hazards models

For time to adoption, we used time-to-event methods. Since this is a time-to-event study, we conducted survival analysis to tailor the site data with right censoring. Right censoring occurs when the study ends before the event has occurred or a subject leaves the study before an event occurs. We created a Kaplan–Meier plot to examine the adoption probability between facilitation groups and then fit Cox proportional-hazards model to estimate the hazard ratio.

For utilization rates in the consult level analysis, we estimated consult completion rates using weighted linear regressions, where the outcome was the site completion rate and the weights were the number of consults at each site. For utilization rates in the site-level analysis, we used linear regressions where sites were not weighted based on the number of consults at each site.

## Results

### Description of study population

Sixty sites were randomized into two facilitation groups. Thirty sites were assigned to the enhanced facilitation group and 30 sites to the basic facilitation group. Sites were evenly distributed among healthcare system networks, varied in the clinical complexity index as defined by the VHA Facility Complexity Model [29], and offered similar prosthetic limb care services (Table 1 and 2). Of the 60 sites included in this study, 36 of the sites (60%) demonstrated complete adoption of FLOW3 during the study period. Among the sites randomized to enhanced facilitation using the intent-to-treat analysis, 21 of 30 (70%) demonstrated complete adoption of FLOW3. Among the sites randomized to basic facilitation, 15 of 30 (50%) demonstrated complete adoption of FLOW3.

**Table 1:** VHA site complexity and number of FLOW3 consults by facilitation group using ITT analysis

Facilitation group	Number of sites	Number of consults	Highest complexity to mid-high complexity (1a–c)	Medium complexity (2)	Low complexity (3)	Polytrauma Amputation Network Site
Enhanced	30	2382	25	2	3	4
Basic	30	2111	21	6	3	3
Total	60	4493	46	8	6	7

**Table 2:** VHA site complexity and number of FLOW3 consults by facilitation group using dose–response analysis

Facilitation group	Number of sites	Number of consults	Highest complexity to mid-high complexity (1a–c)	Medium complexity (2)	Low complexity (3)	Polytrauma Amputation Network Site
Enhanced	18	1843	14	2	2	4
Basic	42	2650	32	6	4	3
Total	60	4493	46	8	6	7

Inclusion in the enhanced facilitation group for the dose–response analysis (attendance of at least two monthly calls) was met by 18 of the 30 sites randomized to the enhanced facilitation group. While 18 sites met the minimum threshold for inclusion in the enhanced facilitation group, 23 sites attended at least 1 of the 6 enhanced facilitation calls, while 11 sites attended 4 or more, and 3 sites attended all 6. Of the 23 sites that attended at least 1 call, the median number of enhanced facilitation calls attended was 3. Using dose–response analysis, 15 of the 18 sites (83%) demonstrated complete adoption of FLOW3, while 21 of 42 sites (50%) of basic facilitation sites demonstrated complete adoption of FLOW3 during the study period.

A description of facility clinical complexity index and the number of consults throughout the study period for the enhanced and basic facilitation groups are summarized in Table 1 and 2. Each consult represents a prescription for prosthetic limb care.

During the study period, the total number of prescriptions entered for prosthetic limb care was 4493, with 2382 entered at sites randomized to the enhanced facilitation group and 2111 entered from sites in the basic facilitation group using the intent-to-treat analysis. Using the dose–response analysis, enhanced facilitation sites entered 1843 consults, and basic facilitation sites entered 2650 consults.

### Time to adoption

#### Time to adoption—intent-to-treat analysis

**Fig. 4:**
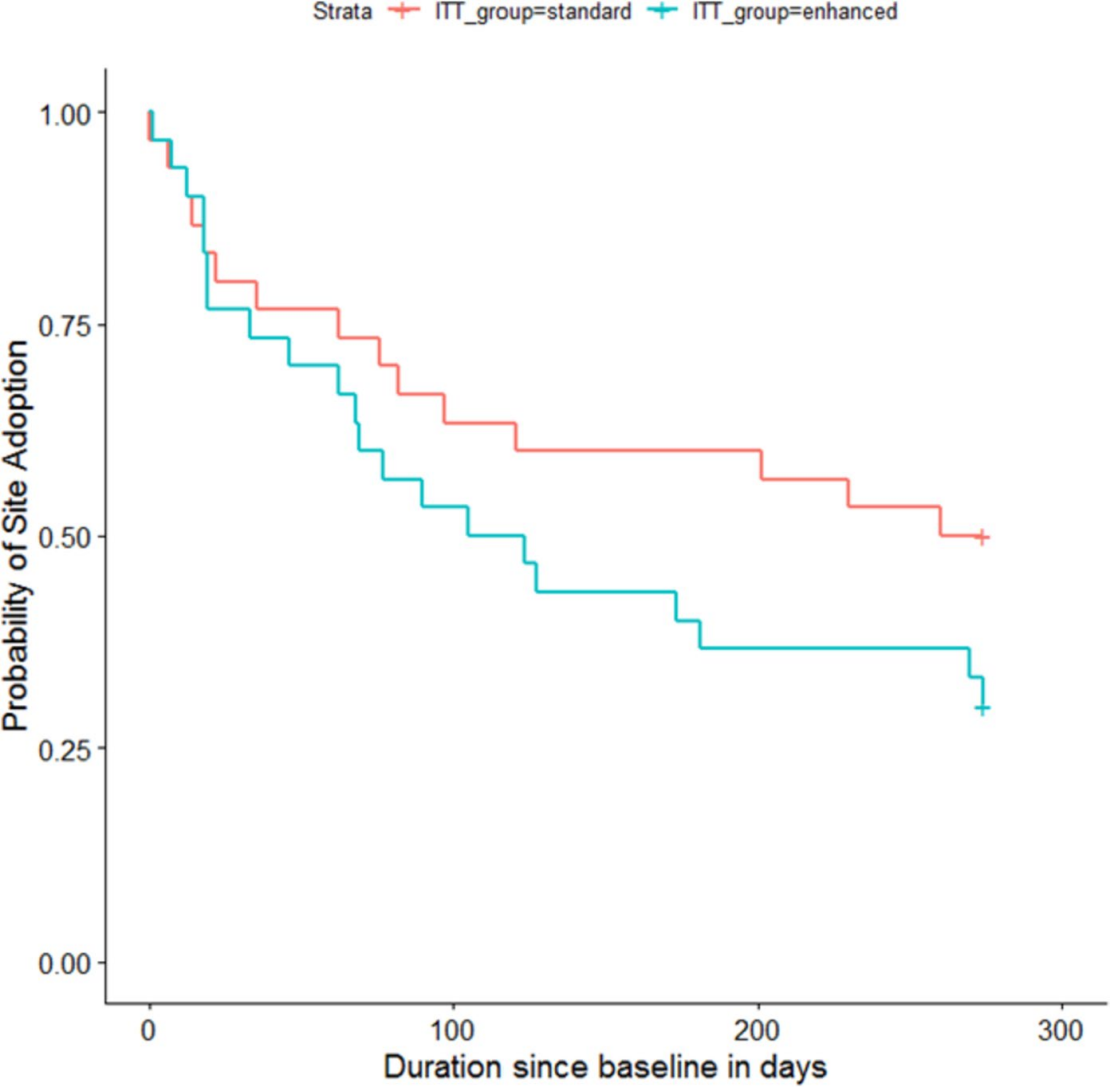
Showing probability of site adoption of FLOW3 plotted against the number of days since the go-live date

Among basic facilitation sites, 15 of 30 sites adopted FLOW3 during the study period. Among enhanced facilitation sites, 21 of 30 sites adopted FLOW3 during the study period. The median time to adoption at sites in the basic facilitation group was 62 days (16, 108). The median time to adoption at sites in the enhanced facilitation group was 68 days (19, 123). We observed wide variability in time to adoption among both basic and enhanced groups. Figure 4 shows a survival estimate for probability of adoption for basic and enhanced groups. The enhanced group was 1.62 times more likely to adopt FLOW3 than basic group (*HR* 1.62, 0.62, 3.14); however, this difference was not statistically significant (*p* = 0.2).

#### Time to adoption—dose–response analysis

**Fig. 5:**
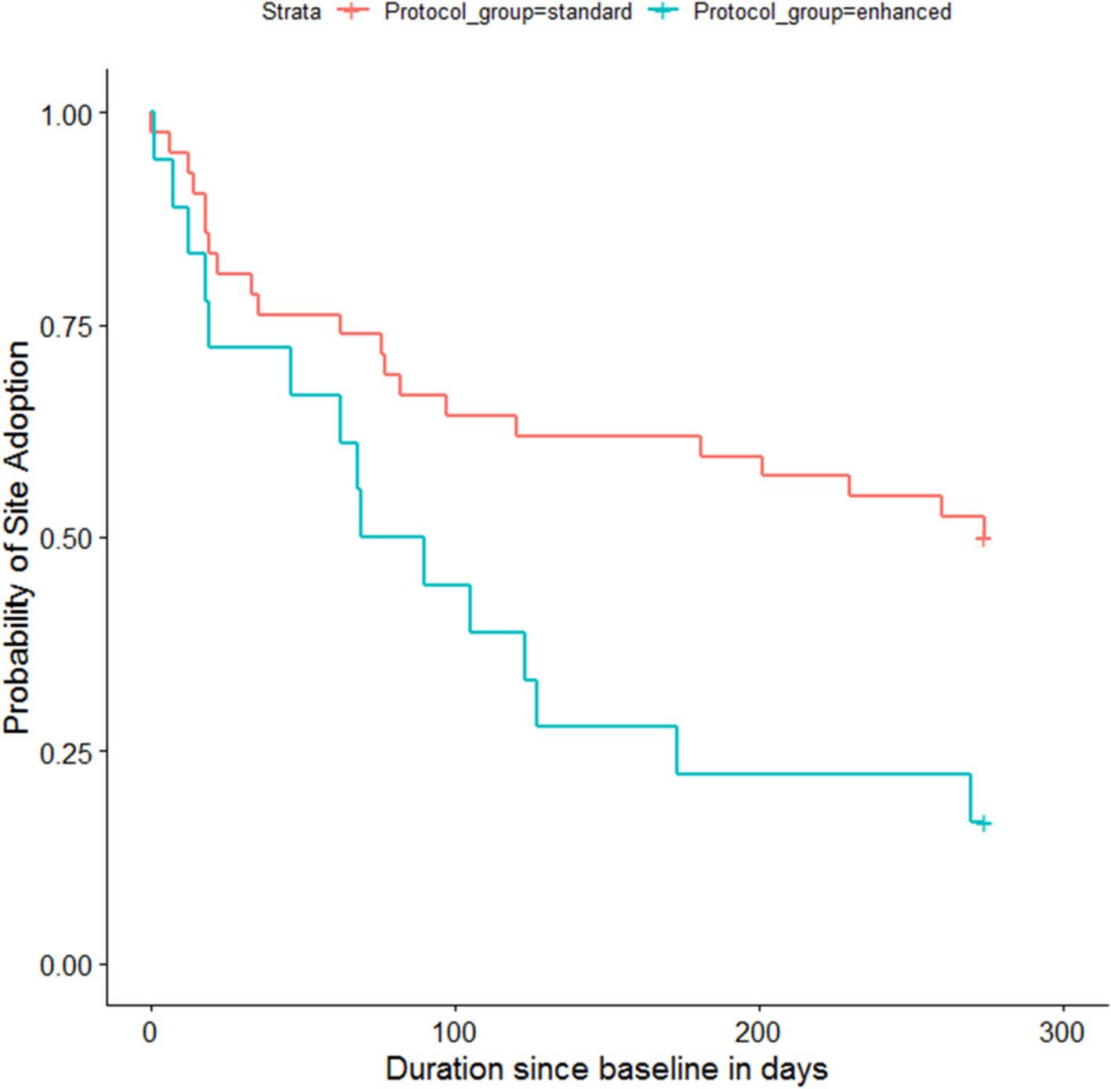
This figure shows the probability of site adoption of FLOW3 plotted against the number of days since the go-live date

Among basic facilitation sites, 21 of 42 sites adopted FLOW3. Among enhanced facilitation sites, 15 of 18 sites adopted FLOW3. The median time to adoption at sites in the basic facilitation group was 62 days (18, 120). The median time to adoption at sites in the enhanced facilitation group was 68 days (18.5, 114). We observed wide variability in time to adoption among both basic and enhanced groups. Figure 5 shows a survival estimate for probability of adoption for basic and enhanced groups. The enhanced group was 2.32 (95% *CI*: 1.18, 4.53) times more likely to adopt FLOW3 than basic group, and this difference was statistically significant (*p* = 0.014).

### Complete utilization rate during implementation period

#### Complete utilization rate during implementation period—ITT analysis

Table 3 indicates that 40% of consults placed at enhanced facilitation sites were identified as “complete,” while only 23% of consults placed at basic facilitation sites were “complete.” The completion rate for FLOW3 consults at sites in the enhanced facilitation group was 0.17 (95% *CI*: 0.31, 0.03) higher compared to sites in the basic group. This difference was statistically significant (*p* = 0.02). The mean completion rate for sites in each the basic facilitation group was 0.1269, and the mean completion rate for sites in the enhanced facilitation group was 0.2405. We observed a 0.11 difference in the consult completion rate (95% *CI*: − 0.007, 0.236) between the basic and enhanced facilitation groups, but this difference was not statistically significant (*p* = 0.07).

**Table 3:** Consult completion rate during implementation period using ITT analysis

Facilitation group	Number of sites	Number of consults	Completion rate
Enhanced	30	1406	0.4018
Basic	30	1283	0.2315
Total	60	2689	0.3206

#### Complete utilization rate during implementation period—dose–response analysis

Table 4 indicates that 38% of consults placed at enhanced facilities were considered “complete,” while almost 28% of consults placed at basic facilities were considered “complete.” The completion rate among consults in the enhanced facilitation group was 0.11 (95% *CI*: 0.26, − 0.05) higher compared to the basic group consults. This difference was not statistically significant (*p* = 0.16). We observed a mean completion rate of 0.1444 for sites in the basic facilitation group and 0.2772 for sites in the enhanced facilitation group. We observed a 0.13 difference in the site completion rate (95% *CI*: 0.28, − 0.01) between the basic and enhanced facilitation groups, but this difference was not statistically significant (*p* = 0.08).

**Table 4:** Consult completion rate during implementation period using dose–response analysis

Facilitation group	Number of sites	Number of consults	Completion rate
Enhanced	18	1093	0.3843
Basic	42	1596	0.2776
Total	60	2689	0.3206

### Complete utilization rate during sustainment period

#### Complete utilization rate during sustainment period—ITT analysis

Table 5 indicates that 48% of consults placed at enhanced facilitation sites were considered “complete,” while only 36% of consults placed at basic facilitation sites were considered “complete.” The completion rate among consults in the enhanced facilitation group was 0.13 (95% *CI*: 0.29, − 0.04) higher compared to the basic group consults. This difference was not statistically significant (*p* = 0.14). The mean completion rate among sites in the basic facilitation group was 0.41 compared to 0.47 among sites in the enhanced facilitation group. We observed a 0.06 difference in the site completion rate (95% *CI*: 0.2741, − 0.1417) between the basic and enhanced facilitation groups, but this difference was not statistically significant (*p* = 0.5).

**Table 5:** Consult completion rate during sustainment period using ITT analysis

Facilitation group	Number of sites	Number of consults	Completion rate
Enhanced	19	951	0.4784
Basic	12	720	0.3569
Total	31	1671	0.4261

#### Complete utilization rate during sustainment period—dose–response analysis

Table 6 indicates that 46% of consults placed at enhanced facilitation sites were considered “complete,” while 40% of consults placed at basic facilitation sites were considered “complete.” The completion rate among consults in the enhanced facilitation group was 0.06 (95% *CI*: 0.23, − 0.11) higher compared to the basic group consults. This difference was not statistically significant (*p* = 0.48). The mean completion rate for sites in the basic facilitation group was 0.44 compared with 0.46 in the enhanced facilitation group. We observed a 0.02 difference in the site completion rate (95% *CI*: 0.2159, − 0.1686) between the basic and enhanced facilitation groups, but this difference was not statistically significant (*p* = 0.06).

**Table 6:** Consult completion rate during sustainment period in dose–response analysis

Facilitation group	Number of sites	Number of consults	Completion rate
Enhanced	14	736	0.4592
Basic	17	935	0.4000
Total	31	1671	0.4261

## Discussion

FLOW3 aimed to leverage ICT to streamline the complex process of providing prosthetic limb care for veterans with limb loss across the VHA. Not all sites implemented FLOW3 during the study period despite planned national rollout. We found that the enhanced facilitation sites were more likely to adopt FLOW3 and had a higher rate of complete utilization during the implementation phase of this study compared to the basic facilitation approach. There were no differences in sustained utilization between the two groups. Strategies used among the enhanced facilitation group, such as customized feedback reports and learning collaborative, can be translated to support adoption and implementation of ICTs in other contexts.

Sites in the enhanced facilitation group received monthly personalized feedback reports and participated in monthly group calls to review site-level performance and work together to troubleshoot issues. Previous studies demonstrate benefits of audit and feedback [30] and suggest that personalized feedback yields better compliance during implementation than team level audit and feedback [31]. Additional work suggests that action plans can enhance the impacts of audit and feedback [32]. Our approach included both team level feedback and personalized reports with action plans. We were unable to track how the site-specific performance feedback reports received by sites in the enhanced facilitation group were utilized, but several sites initiated email communication for help troubleshooting with the implementation team after receiving their reports.

The monthly group calls offered to sites in the enhanced facilitation group created a learning collaborative where sites could review performance, learn from sites that had previously implemented, and work together to overcome challenges with adoption and implementation. Previous work suggests that learning collaboratives enhance implementation of interventions through fostering a culture that supports use of evidence-based practices through modeling and imitation of colleagues who have been successful [33, 34]. Only 3 sites attended all 6 calls, and the median number of calls attended among the 23 sites that attended at least 1 call was 3. Despite low attendance on calls, these sites still demonstrated improved adoption using the dose–response analysis and better utilization outcomes using ITT analysis during the implementation period compared with basic facilitation sites. This suggests that the benefit of the monthly calls could have been achieved with fewer calls (a lower dose) and less time investment from the implementation team. It is important to consider the necessary dose of facilitation to support implementation of frontline developed ICTs like FLOW3. The FLOW3 implementation team was comprised of the clinicians and staff who developed FLOW3, all of whom supported implementation in addition to their clinical and administrative roles. It is often the case that time spent supporting implementation competes with other demands in a busy healthcare system [35], and so understanding the “optimal” dose of facilitation may ameliorate time pressures during implementation.

Adoption, implementation, and sustainment of ICT are critical to realizing their benefits. We observed that sites in the enhanced facilitation group were more likely to adopt FLOW3 in the dose response analysis and had a higher rate of complete utilization during the implementation period in the intent to treat analysis. These differences were likely supported by the benefits of the enhanced protocol discussed above. However, assignment of sites to the enhanced facilitation group in the dose–response analysis was based on strict criteria, and we are unable to say whether sites that adhered to those criteria were different from other sites in important ways that were not captured in our analysis. For example, this study did not account for potential differences in site context that may influence adoption and implementation. A rigorous pre-implementation evaluation of each potential FLOW3 site was not feasible given the timeline and resources available for this study, but future work on factors influencing ICT implementation would benefit from a mixed-method evaluation using an implementation framework (e.g., [36]) to assess factors like local processes or attitudes that may enhance or inhibit implementation, leadership support and engagement, and organizational readiness to change. Results of such work could help identify impactful and feasible ways to engage sites and busy staff and clinicians in the implementation process to promote ICT uptake and utilization.

We did not find a statistically significant difference in complete FLOW3 utilization rate during the sustainment period, but the higher utilization rate seen among enhanced facilitation sites in the dose–response analysis approaches statistical significance. Future work should assess sustained use of FLOW3 over a longer period and identify strategies to promote sustainment. For example, automated reporting on site-level utilization, ongoing monitoring through use of a dashboard, or periodically sharing site level, patient feedback on the limb acquisition process could incentivize sustained utilization. FLOW3 affects the care process at multiple points and must be utilized completely for the greatest improvements in patient experience. The FLOW3 process sets standards for team-based clinical decision-making due to requirements for an accurate prosthesis prescription entered by the physician that reduces any vagueness or discrepancy that may lead to delays from back-and-forth conversation between VA and non-VA providers involved in prosthetic limb care. An accurate prescription further ensures a clearly described HCPCS list which correlates to the payment system between the government and its vendors. Finally, this system developed a process to monitor use and timeliness of each step and facilitate efficient practices while maintaining compliance with VHA directives and following best practice guidelines for patient care.

### Limitations

Understanding which element of enhanced facilitation had the greatest impact on adoption, implementation, and sustainment was beyond the scope of this study and warrants future research. Additionally, we did not collect contextual information on the sites included in the study to understand important differences (e.g., departmental culture or staffing) that may have impacted participation in enhanced facilitation activities or influenced willingness to adopt and implement a new process. Further, sites that were more likely to adhere to the enhanced facilitation protocol may have been higher performing sites in general, explaining their higher performance in the outcome of interest. Finally, due to constraints of the project timeline, we only followed sites for 3 months during the sustainment period (versus 6 months during the implementation period). Determining if benefits of the enhanced facilitation protocol were sustained over a longer period would be informative.

## Conclusions

It is critical to understand adoption, implementation, and sustainment of new innovations. Our study suggests that learning collaborative calls and individualized performance feedback reports can improve timeliness of adoption and complete utilization during some periods of study for novel ICT. This study was not able to assess how performance feedback reports were used and did not evaluate stakeholder satisfaction with implementation and use of FLOW3. Future work should assess how to better engage sites to promote adoption and implementation and ultimately sustainment.

## Data Availability

Data are available from the authors upon reasonable request.

## Abbreviations

ICT: Information and communication technologies
VHA: Veterans Health Administration
VA: Department of Veteran’s Affairs
HCPCS: Healthcare Common Procedure Coding System
VISN: VA Integrated Service Network
ITT: Intent to treat
PP: Per protocol
ASoC: VA Amputation System of Care
QUERI: Quality Enhancement Research Initiative
DOE: VHA Diffusion of Excellence
RPS: VHA Rehabilitation and Prosthetic Services

## References

[1] Westbrook JI (2005). Do online information retrieval systems help experienced clinicians answer clinical questions?. JAMIA.

[2] Verbeke F, Karara G, Nyssen M (2013). Evaluating the impact of ICT-tools on health care delivery in sub-Saharan hospitals. Stud HealthTechnol Inform.

[3] Rouleau G, Gagnon MP, Côté J, Payne-Gagnon J, Hudson E, Dubois CA (2017). Impact of information and communication technologies on nursing care: results of an overview of systematic reviews. J Med Internet Res.

[4] Gagnon MP, Desmartis M, Labrecque M, Car J, Pagliari C, Pluye P (2012). Systematic review of factors influencing the adoption of information and communication technologies by healthcare professionals. J Med Syst.

[5] Gagnon MP, Légaré F, Labrecque M, Frémont P, Pluye P, Gagnon J, et al. Interventions for promoting information and communication technologies adoption in healthcare professionals. Cochrane Effective Practice and Organisation of Care Group, editor. Cochrane Database of Systematic Reviews. 2009; Available from: http://doi.wiley.com/10.1002/14651858.CD006093.pub2. [Cited 2021 Jun 8]. 10.1002/14651858.CD006093.pub2 PMC3973635 19160265

[6] Ovretveit J, Scott T, Rundall TG, Shortell SM, Brommels M (2007). Improving quality through effective implementation of information technology in healthcare. IJQHC.

[7] Gagnon MP, Desmartis M, Labrecque M, Car J, Pagliari C, Pluye P (2012). Systematic review of factors influencing the adoption of information and communication technologies by healthcare professionals. J Med Syst.

[8] Gururajan R, Hafeez-Baig A (2014). An empirical study to determine factors that motivate and limit the implementation of ICT in healthcare environments. BMC Med Inform Decis Mak.

[9] Callen JL, Braithwaite J, Westbrook JI (2007). Cultures in hospitals and their influence on attitudes to, and satisfaction with, the use of clinical information systems. Soc Sci Med.

[10] Dutta B, Hwang HG (2020). The adoption of electronic medical record by physicians: a PRISMA-compliant systematic review. Medicine.

[11] Gosling AS (2003). Clinical team functioning and IT innovation: a study of the diffusion of a point-of-care online evidence system. JAMIA.

[12] Audit of the Management and Acquisition of Prosthetic Limbs. 2012. Available from: va.gov/oig/pubs/VAOIG-11–02254–102.pdf. [Cited 2021 Jun 9].

[13] Department of Veterans Affairs, Office of Inspector General. Healthcare inspection prosthetic limb care in VA facilities. Report No.: 11–02138–116. Available from: https://www.va.gov/oig/pubs/VAOIG-11-02138-116.pdf

[14] Bekkering GE, van Tulder MW, Hendriks EJ, Koopmanschap MA, Knol DL, Bouter LM (2005). Implementation of clinical guidelines on physical therapy for patients with low back pain: randomized trial comparing patient outcomes after a standard and active implementation strategy. Phys Ther.

[15] Becker A, Leonhardt C, Kochen MM, Keller S, Wegscheider K, Baum E (2008). Effects of two guideline implementation strategies on patient outcomes in primary care: a cluster randomized controlled trial. Spine.

[16] Beidas RS, Edmunds JM, Marcus SC, Kendall PC (2012). Training and consultation to promote implementation of an empirically supported treatment: a randomized trial. PS.

[17] Kilbourne AM, Almirall D, Eisenberg D, Waxmonsky J, Goodrich DE, Fortney JC (2014). Protocol: Adaptive Implementation of Effective Programs Trial (ADEPT): cluster randomized SMART trial comparing a standard versus enhanced implementation strategy to improve outcomes of a mood disorders program. Implement Sci.

[18] Kilbourne AM, Abraham KM, Goodrich DE, Bowersox NW, Almirall D, Lai Z (2013). Cluster randomized adaptive implementation trial comparing a standard versus enhanced implementation intervention to improve uptake of an effective re-engagement program for patients with serious mental illness. Implement Sci.

[19] Chinman M, Goldberg R, Daniels K, Muralidharan A, Smith J, McCarthy S (2021). Implementation of peer specialist services in VA primary care: a cluster randomized trial on the impact of external facilitation. Implement Sci.

[20] Chinman M, Daniels K, Smith J, McCarthy S, Medoff D, Peeples A (2017). Provision of peer specialist services in VA patient aligned care teams: protocol for testing a cluster randomized implementation trial. Implement Sci.

[21] Colquhoun HL, Brehaut JC, Sales A, Ivers N, Grimshaw J, Michie S (2013). A systematic review of the use of theory in randomized controlled trials of audit and feedback. Implement Sci.

[22] Glasgow RE, Vogt TM, Boles SM (1999). Evaluating the public health impact of health promotion interventions: the RE-AIM framework. Am J Public Health.

[23] Stetler CB, Legro MW, Rycroft-Malone J, Bowman C, Curran G, Guihan M (2006). Role of “external facilitation” in implementation of research findings: a qualitative evaluation of facilitation experiences in the Veterans Health Administration. Implement Sci.

[24] Ranganathan P, Pramesh CS, Aggarwal R (2016). Common pitfalls in statistical analysis: intention-to-t=Tt versus per-protocol analysis. Perspect Clin Res.

[25] Sedgwick P (2015). Intention to treat analysis versus per protocol analysis of trial data. BMJ.

[26] Porta N, Bonet C, Cobo E (2007). Discordance between reported intention-to-treat and per protocol analyses. J. Clin. Epidemiol.

[27] Webster J, Scholten J, Young P, Randolph BJ (2020). Ten-year outcomes of a systems-based approach to longitudinal amputation care in the US Department of Veteran Affairs. Fed Pract.

[28] PROGRAM GUIDE: 1200.21 VHA operations activities that may constitute research. Available From: ProgramGuide-1200–21-VHA-Operations-Activities.pdf(va.gov) . [Cited June 29, 2023].

[29] National Academies of Sciences E, Education D of B and SS and, Integration B on HS, Sciences D on E and P, Environment B on I and the C, Administration C on FSR for VH. Nature of Veterans Health Administration Facilities Management (Engineering) Tasks and Staffing. Facilities Staffing Requirements for the Veterans Health Administration-Resource Planning and Methodology for the Future. National Academies Press (US); 2019. Available from: https://www.ncbi.nlm.nih.gov/books/NBK555777/. [Cited 2023 Feb 13]. 32293829

[30] Audit and feedback: effects on professional practice and healthcare outcomes - Ivers, N - 2012 | Cochrane Library. Available from: https://www.cochranelibrary.com/cdsr/doi/10.1002/14651858.CD000259.pub3/full. [Cited 2022 Sep 23]. 10.1002/14651858.CD000259.pub3 PMC11338587 22696318

[31] Borgert M, Binnekade J, Paulus F, Goossens A, Vroom M, Dongelmans D (2016). Timely individual audit and feedback significantly improves transfusion bundle compliance—a comparative study. IJQHC.

[32] Gardner B, Whittington C, McAteer J, Eccles MP, Michie S (2010). Using theory to synthesise evidence from behaviour change interventions: the example of audit and feedback. Soc Sci Med.

[33] Adams S, Titler MG (2010). Building a learning collaborative. Worldviews Evid-Based Nurs.

[34] Solomon DH, Losina E, Lu B, Zak A, Corrigan C, Lee SB (2017). Implementation of treat-to-target in rheumatoid arthritis through a learning collaborative: results of a randomized controlled trial. Arthritis & Rheumatology.

[35] Li SA, Jeffs L, Barwick M, Stevens B (2018). Organizational contextual features that influence the implementation of evidence-based practices across healthcare settings: a systematic integrative review. Syst Rev.

[36] Feldstein AC, Glasgow RE. A practical, robust implementation and sustainability model (PRISM) for integrating research findings into practice. Jt Comm J Qual Patient Saf. 2008;34(4):228-43. 10.1016/s1553-7250(08)34030-6 18468362

